# Differential expression of neuregulin-1 isoforms and downregulation of erbin are associated with Erb B2 receptor activation in diabetic peripheral neuropathy

**DOI:** 10.1186/2051-5960-1-39

**Published:** 2013-07-17

**Authors:** Pan Pan, Rick T Dobrowsky

**Affiliations:** 1 Department of Pharmacology and Toxicology, University of Kansas, Lawrence, KS, USA

**Keywords:** Nerve conduction velocity, Erlotinib, Sensory hypoalgesia, Intra epidermal nerve fibers, Sural nerve

## Abstract

**Background:**

Aberrant neuron/glia interactions can contribute to a variety of neurodegenerative diseases and we have previously demonstrated that enhanced activation of Erb B2, which is a member of the epidermal growth factor receptor (EGFR) family, can contribute to the development of diabetic peripheral neuropathy (DPN). In peripheral nerves, Erb B receptors are activated by various members of the neuregulin-1 (NRG1) family including NRG1 Type I, NRG1 Type II and NRG1 Type III to regulate Schwann cell (SC) growth, migration, differentiation and dedifferentiation. Alternatively, Erb B2 activity can be negatively regulated by association with the Erb B2-interacting protein, erbin. Since the effect of diabetes on the expression of NRG1 isoforms and erbin in peripheral nerve are unknown, the current study determined whether changes in NRG1 isoforms and erbin may be associated with altered Erb B2 signaling in DPN.

**Results:**

Swiss Webster mice were rendered diabetic with streptozotocin (STZ) and after 12 weeks of diabetes, treated with erlotinib, an inhibitor of Erb B2 activation. Inhibition of Erb B2 signaling partially reversed several pathophysiologic aspects of DPN including a pronounced sensory hypoalgesia, nerve conduction velocity deficits and the decrease in epidermal nerve fiber innervation. We also observed a decrease of NRG1 Type III but an increase of NRG1 Type I level in diabetic sural nerves at early stage of diabetes. With disease progression, we detected reduced erbin expression and enhanced MAPK pathway activity in diabetic mice. Inhibition of Erb B2 receptor suppressed MAPK pathway activity in treated-diabetic sural nerves.

**Conclusions:**

These results support that hyperglycemia may impair NRG1/Erb B2 signaling by disrupting the balance between NRG1 isoforms, decreasing the expression of erbin and correspondingly activating the MAPK pathway. Together, imbalanced NRG1 isoforms and downregulated erbin may contribute to the dysregulation of Erb B2 signaling in the development of DPN.

## Background

Diabetic peripheral neuropathy (DPN) is one of the most prevalent complications of chronic diabetes and is the leading cause of non-traumatic lower limb amputation [1]. DPN patients often present with spontaneous positive (burning, itching, abnormal sensation to temperature, pain) or negative (numbness, insensitivity) sensory symptoms that are observed in a distal-to-proximal manner [2]. DPN patients often exhibit a reduction in nerve conduction velocity, loss of nerve fibers and segmental demyelination [2,3]. Mechanistically, the development of DPN has multiple contributing factors. Hyperglycemia induces oxidative stress and alters several metabolic and vascular pathways that contribute to an increase in formation of advanced glycation end-products, excessive polyol synthesis, enhanced protein kinase C activation and an increase in poly-ADP ribose polymerase activity [4]. Another potential mechanism is altered neurotrophism since hyperglycemia can damage peripheral nerves by changing the expression, proteolytic cleavage, axonal transport and function of growth factors. For instance, diabetic rats showed decreased retrograde axonal transport of nerve growth factor (NGF) and brain-derived neurotrophic factor (BDNF), members of the neurotrophin family [5]. In addition, insulin-like growth factors affect neuronal survival, growth and regeneration and are decreased in nerve of diabetic rats [6]. Neuregulin 1 (NRG1) is a growth factor that interacts with Erb B receptors, which localize primarily to Schwann cells (SC), and is critical for both SC differentiation and degeneration. However, little is known about how diabetes may affect gliotrophic signaling by altering NRG1 levels and Erb B2 signaling in the peripheral nervous system (PNS).

The NRG1 family comprises more than 30 membrane-bound and secreted proteins generated by alternative promoter usage and extensive RNA splicing [7]. The NRG1 family is subdivided into six major isoforms based on distinct amino termini but NRG1 Types I, II, and III are the best characterized in the peripheral nervous system. However, all NRG1 family members have an epidermal growth factor (EGF)-like domain which is sufficient and necessary to activate the Erb B members of the EGF receptor (EGFR) family [8]. The EGFR family contains four members, EGFR, Erb B2, Erb B3 and Erb B4. Erb B2 is a ligand-less receptor and Erb B3 has no tyrosine kinase activity. In adult SCs, NRG1 binding to Erb B3 induces formation of primarily Erb B2 and Erb B3 heterodimers [9]. Receptor dimerization activates the intrinsic tyrosine kinase activity of Erb B2 which initiates downstream signaling pathways.

NRG1/Erb B signaling guides every developmental stage of the SC lineage, such as promoting the gliogenic fate of neural crest cells, migration of SC precursors and subsequent proliferation, survival, and differentiation [8,10]. Physiologically, the axonal expression of NRG1 Type III is necessary to promote myelination and regulate myelin thickness *in vivo*[11,12]. Moreover, disruption of Erb B signaling by transgenic overexpression of a dominant-negative Erb B4 receptor resulted in abnormal myelin formation, disrupted Remak bundle structure, and neuropathic changes in both myelinated [13] and unmyelinated [14] sensory fibers. Interestingly, pathologic activation of Erb B2 following axotomy can also promote SC dedifferentiation and demyelination [15]. Though NRG1 Type III promotes myelination, NRG1 Type I and NRG1 Type II have been shown to induce demyelination *in vitro*[16] and this dedifferentiation response is tightly linked to the activation of the p42/p44 MAPK pathway [17,18]. Therefore, changes in the expression of isoforms of NRG1 may provide one mechanism for activation of Erb B2 and p42/p44 MAPK signaling in pathophysiologic states linked to neuropathy and myelin degeneration. Furthermore, Erb B2 is also negatively regulated by the Erb B2-interacting protein (erbin), a downstream adapter protein that specifically interacts with Erb B2, but not Erb B3 or Erb B4 [19]. Erbin is mostly expressed in regions where myelination is abundant and erbin null mice have hypomyelinated peripheral nerves, suggesting it is necessary for normal myelin thickness of peripheral nerves [20]. Following recruitment to Erb B2, erbin can inhibit the p42/p44 MAPK pathway and potentially antagonize NRG1-induced demyelination.

We have previously demonstrated that a transient increase in Erb B2 receptor activation contributed to diabetes-induced sensory neuropathy since inhibiting Erb B2 signaling reversed some of the pathophysiologic features of DPN [21]. Therefore, disrupted Erb B2 signaling might contribute to the axonal degeneration and SC demyelination in diabetic nerves. However, it remains unknown whether the increase in Erb B2 activity in diabetic nerves is associated with changes in NRG1 isoforms and erbin that may serve as positive and negative regulators of Erb B2 signaling, respectively.

To elucidate the role of altered NRG1/Erb B2 signaling in DPN, we used diabetic Swiss Webster mice, which after prolonged diabetes develop severe pathophysiologic symptoms of DPN [22]. We report that diabetes altered the expression of NRG1 Type III and NRG1 Type I in distal nerve fibers with increasing duration of diabetes. In particular, a decrease in NRG1 Type III expression was accompanied by an increased expression of NRG1 Type I level in diabetic sural nerves. Additionally, the levels of erbin were decreased in sciatic nerves of diabetic mice and this corresponded with an increase in p42/p44 MAPK pathway activity. These data are the first to characterize that diabetes can alter the expression of proteins that serve as positive and negative regulators of Erb B2 activity and suggest that an altered neuregulinism may contribute to SC pathology in DPN.

## Results

### Erb B2 receptor inhibitor attenuates pathophysiological indices of DPN

**Figure 1:**
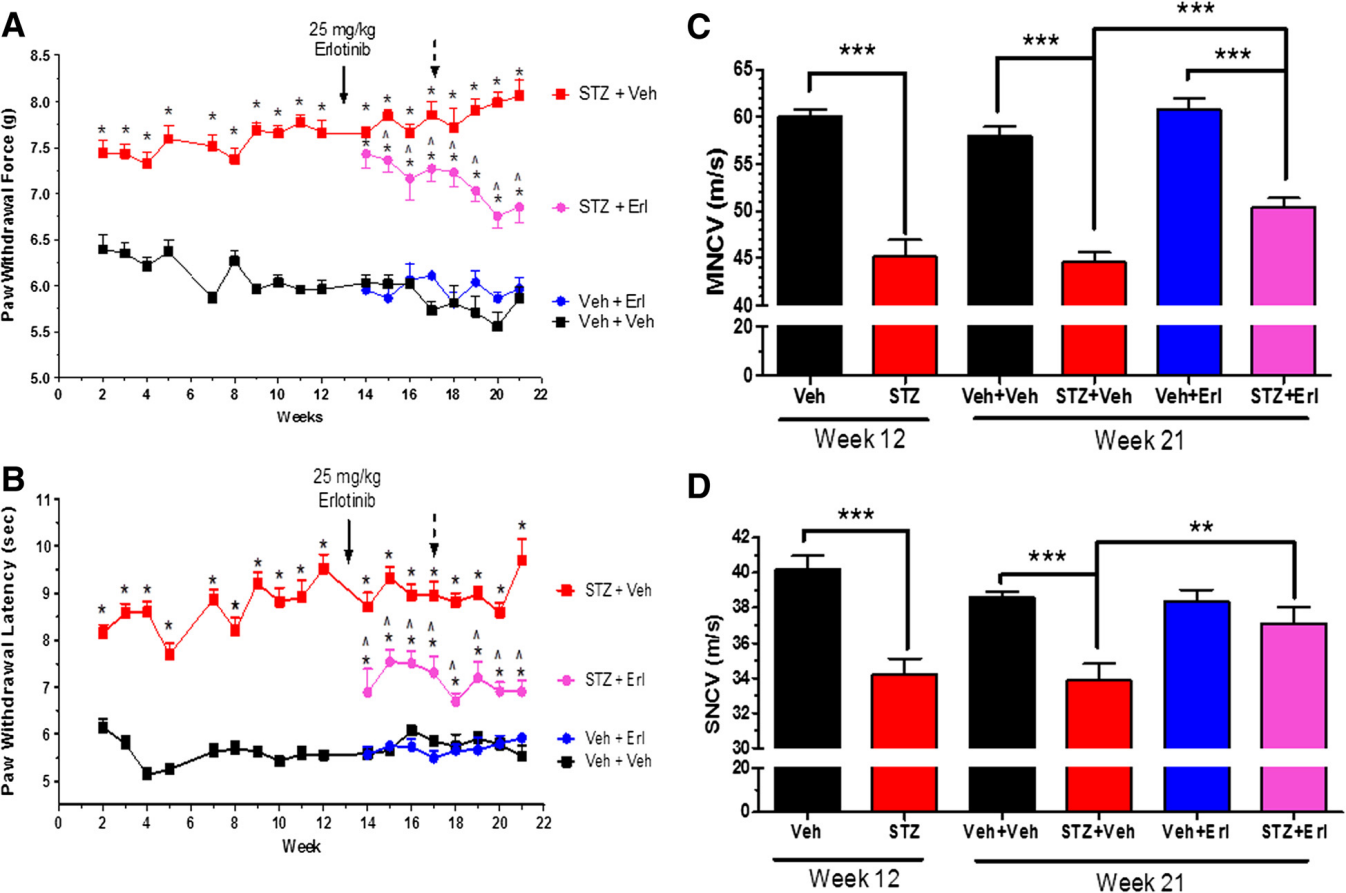
**Inhibition of Erb B2 attenuated NCV and sensory deficits in diabetic mice.** Swiss Webster mice were rendered diabetic and after two weeks of diabetes, mechanical **(A)** and thermal sensitivity **(B)** were assessed weekly (*, p < 0.05 versus time-matched Veh + Veh; ^, p < 0.05 versus time-matched STZ + Veh). Assessment of MNCV **(C)** and SNCV **(D)** in a subgroup of animals (n = 4 per group) after 12 weeks of diabetes confirmed the onset of nerve dysfunction prior to drug treatment. At 13 weeks of diabetes, animals were given vehicle or 25 mg/kg erlotinib once per week for 4 weeks (solid arrow) and then twice per week (dashed arrow) for the final four weeks (n = 8-12 per group). Erlotinib significantly improved mechanical and thermal sensitivity and decreased the deficits in both MNCV and SNCV compared to vehicle treated diabetic mice (**, p < 0.01; ***, p < 0.001).

Swiss Webster mice were rendered diabetic with STZ and changes in mechanical and thermal sensitivity were monitored for 12 weeks to assess the onset of hypoalgesia in both myelinated (mechanical) and unmyelinated (thermal) nerve fibers. Diabetes induced a clear mechanical (Figure 1A) and thermal (Figure 1B) hypoalgesia and after 12 weeks this was associated with a significant decrease in both motor (MNCV) and sensory (SNCV) nerve conduction velocity compared to non-diabetic animals (Figure 1C and D). To address whether Erb B2 signaling contributed to the sensory deficits, mice were treated at week 13 with vehicle or 25 mg/kg erlotinib, which is a clinically approved inhibitor of the EGFR that can also inhibit Erb B2 receptors [21]. The mice were initially given one treatment per week for 4 weeks and this modestly improved mechanical and thermal sensitivity. Increasing the frequency of dosing to twice per week (Figure 1A and B, dashed arrows) yielded a greater improvement in mechanical sensitivity, but thermal sensitivity did not change beyond that seen with a single dose per week. After 8 weeks of erlotinib treatment, the pre-existing deficits in both MNCV and SNCV were partially reversed (Figure 1C and D). Interestingly, the improvement in SNCV and thermal hypoalgesia in erlotinib-treated diabetic Swiss Webster mice was not observed in our previous study using C57Bl/6 mice [21]. Whether this observation is attributable to differences between the mouse strains is unclear. Nonetheless, these data support that Erb B2 signaling may contribute to glial cell dysfunction in DPN.

**Figure 2:**
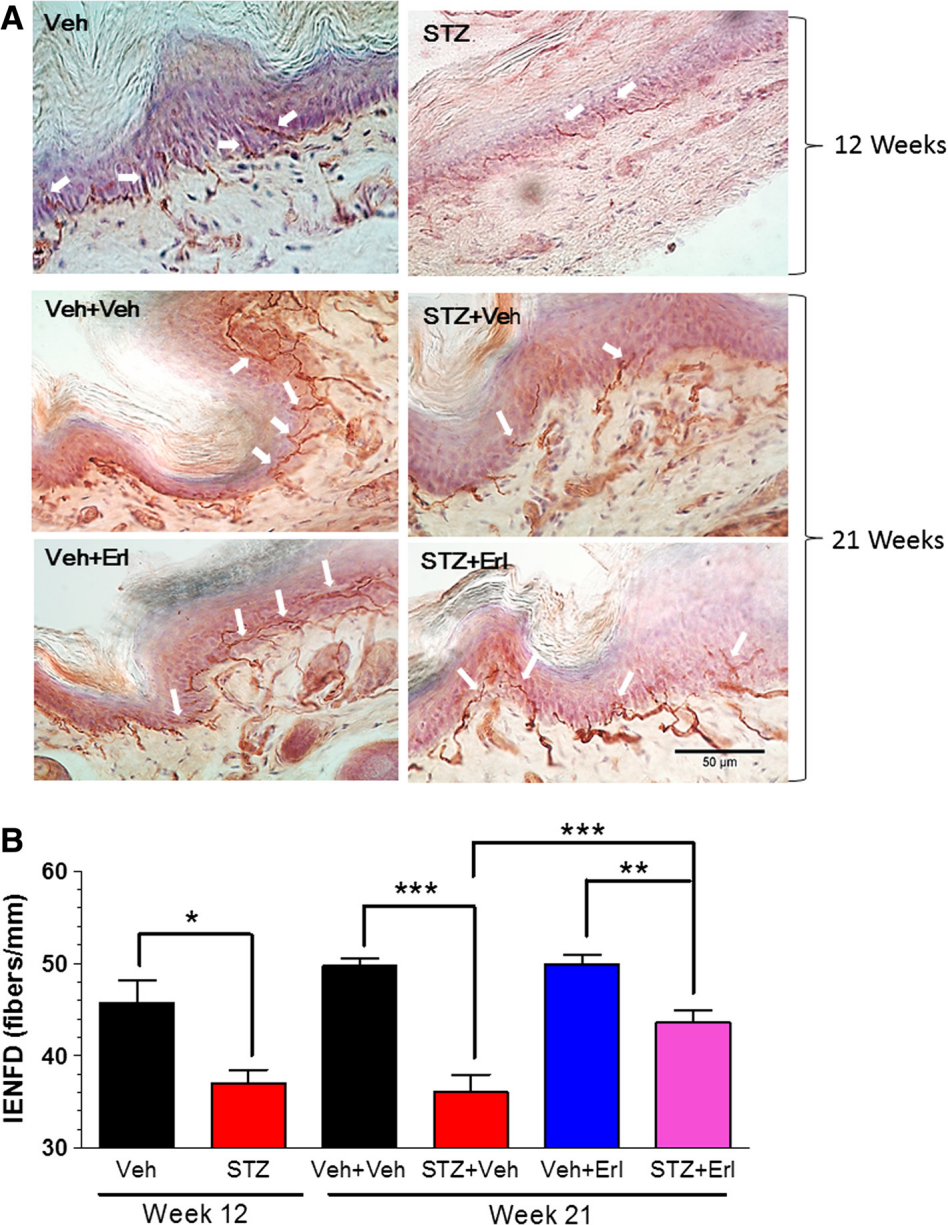
**Inhibition of Erb B2 improved IENFD in DPN mice.** Footpad samples were collected from the plantar surface of the hind paws after 12 (n = 4 per group) or 21 weeks (n = 6–8 per group) of treatment. **(A)**: Representative images of IENFD in non-diabetic (Veh) and diabetic (STZ) mice treated with vehicle (Veh) or erlotinib (Erl). Nerve fibers immunopositive for PGP 9.5 are stained red by the chromagen (arrows) and epidermal cells were stained purple by hematoxylin. **(B)**: Quantification demonstrated a significant loss of nerve innervation in diabetic mice prior to drug administration and erlotinib treatment induced a partial recovery in fiber loss (*, p < 0.05; **, p < 0.01; ***, p < 0.001).

Assessing changes in intraepidermal nerve fiber density (IENFD) is emerging as a powerful technique for accurately diagnosing and staging the onset of a small fiber neuropathy in patients [23] and animal models [24,25]. We processed the plantar surface of the hind paws and stained the nerve fibers with an antibody against protein gene product 9.5 (PGP 9.5), which is a cytosolic ubiquitin C-terminal hydroxylase specifically expressed in neurons. The PGP 9.5-positive nerve fibers that crossed the epidermal/dermal junction were counted to quantify distal nerve innervation (Figure 2A and B). As anticipated, 12 weeks of diabetes significantly decreased IENFD but fiber loss was not significantly greater after 21 weeks of diabetes. However, erlotinib treatment partially reversed the diabetes-induced loss of the largely unmyelinated intra-epidermal nerve fibers. Though the increase in IENFD in erlotinib-treated diabetic mice may contribute to the increased sensitivity to noxious thermal stimuli, improved thermal sensitivity can occur in the absence of an increase in epidermal fibers [25,26].

After 12 and 21 weeks of diabetes, fasting blood glucose (FBG) levels and HbA1c were increased. As expected, diabetic mice showed a loss of body weight compared to age-matched, vehicle-treated control mice (Table 1) and erlotinib treatment did not affect FBG or body weight compared to vehicle-treated diabetic mice. These results indicate that the effect of erlotinib on improving the diabetes-induced nerve deficits in both myelinated and unmyelinated nerve fibers were not related to correction of the systemic hyperglycemia, consistent with our previous observation in diabetic C57Bl/6 mice [21].

**Table 1:** Body weights, FBG and HbA1c values from the Swiss Webster mice

**Week**	**Treatment**	**FBG (mg/dl)**	**Weight (g)**	**(n)**	**HbA1c (%)**	**(n)**
Week 12	Veh	112 ± 21	37.2 ± 5.4	10	5.0 ± 0.4	6
	STZ	595 ± 12*	30.6 ± 3.7*	11	12.9 ± 0.2*	7
Week 21	Veh + Veh	121 ± 26	42.6 ± 5.4	8	5.1 ± 0.1	3
	Veh + Erl	104 ± 17	43.4 ± 2.7	8	5.0 ± 0.2	3
	STZ + Veh	545 ± 106*	34.5 ± 3.5*	8	10.2 ± 1.7*	4
	STZ + Erl	584 ± 40^#^	37.0 ± 5.5^#^	6	11.4 ± 1.1^#^	4

*, p < 0.05 vs. Veh or Veh + Veh; #, p < 0.05 vs. Veh + Erlotinib (Erl).

### Diabetes differentially alters the expression of NRG1 isoforms in sural nerve

**Figure 3:**
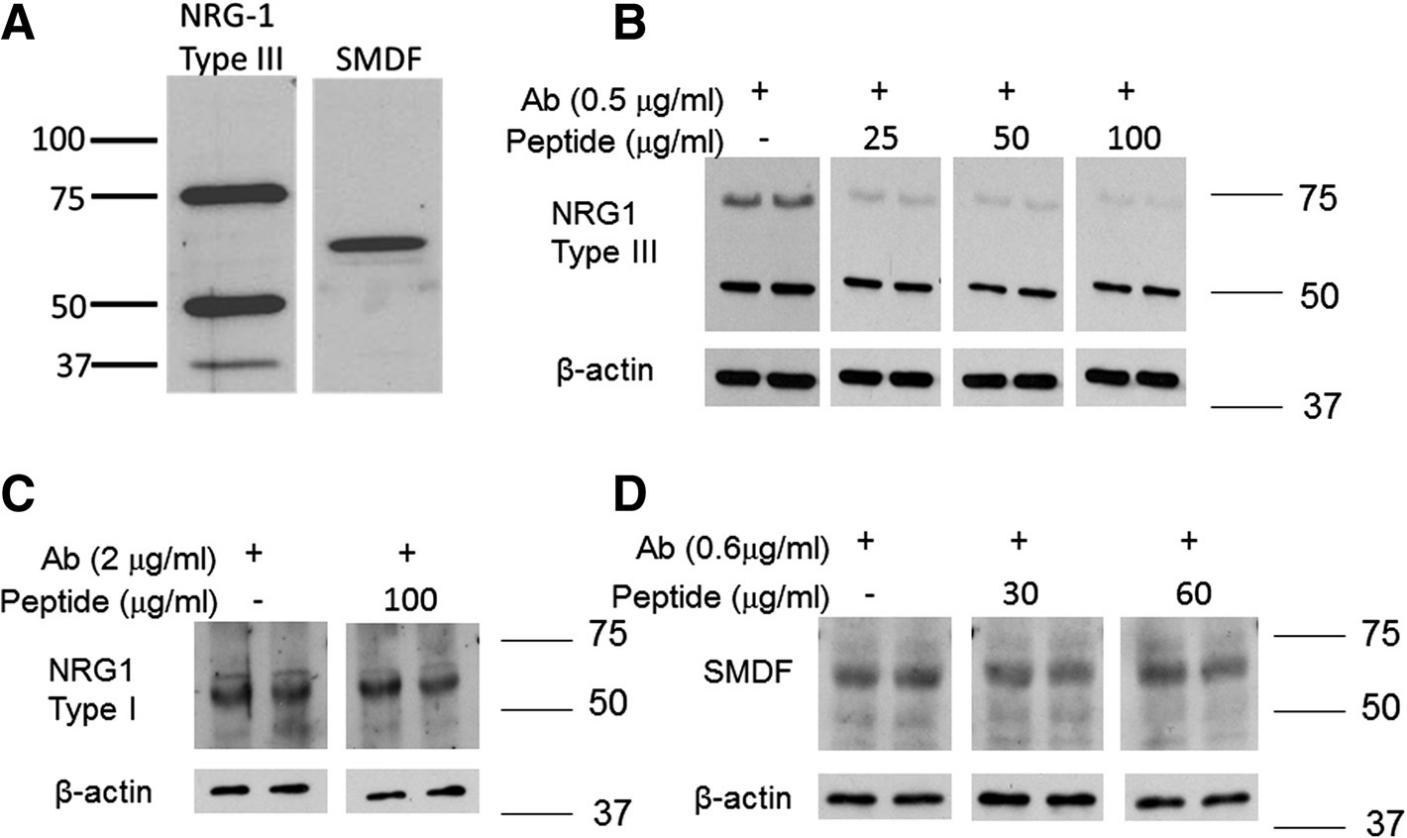
**Validation of specificity of the custom NRG1 Type III antibody. (A)**: Comparison of NRG1 Type III detection using the custom N-terminal NRG1 Type III polyclonal antibody (left) and a commercial C-terminal NRG1 Type III polyclonal antibody (SMDF, right). **(B**-**D) **Demonstration of the specificity of the N-terminal NRG1 Type III polyclonal antibody. The indicated amounts of the N-terminal NRG1 Type III antibody **(B)**, a polyclonal antibody against NRG1 Type I **(C) **or the C-terminal NRG1 Type III antibody (SMDF, D) were preabsorbed with the immunizing peptide in a 1:50 to 1:200 ratio for 1 hr at 25°C and then used for immunoblot analysis of a sciatic nerve sample. The peptide specifically decreased the detection of the 75 kDa NRG1 Type III band but had no effect on detecting NRG1 Type I or the 65 kDa C-terminal NRG1 Type III fragment.

Though Erb B2 activity can contribute to DPN, it is unknown whether this may be associated with changes in the expression of NRG1 isoforms which can promote SC differentiation or dedifferentiation. Sciatic and sural nerves were isolated from control and diabetic mice, protein lysates were separated by SDS-PAGE and probed with two different NRG1 Type III antibodies. Using a previously reported strategy [12], we raised a polyclonal antibody against a peptide sequence near the distinct cysteine-rich domain (CRD) in the NRG1 Type III isoform. Immunoblot of sciatic nerve extracts with this antibody detected a prominent band at about 75 kDa (Figure 3A). This band represents the N-terminal fragment after cleavage and is consistent with the protein size previously reported [27]. As a complementary approach, we also used a well characterized commercial NRG1 Type III antibody that was raised against a region within the C-terminal domain of NRG1 Type III (SMDF, sensory and motor neuron derived factor) and detects a band at about 65 kDa.

To confirm antibody specificity, 0.5 μg/ml of the NRG1 Type III antibody was preabsorbed with the immunizing peptide in a 1:50 to 1:200 mass ratio prior to immunoblot analysis of sciatic nerve lysates (Figure 3B). The immunizing peptide decreased the amount of NRG1 Type III present at 75 kDa but had no effect on the band present at 50 kDa, indicating it is non-specifically recognized by the antibody. Though the identity of the minor 37 kDa band in Figure 3A is unknown, it may represent a degradation product of NRG1 Type III as it was not consistently detected. Importantly, preabsorbing the peptide to an antibody against NRG1 Type I (Figure 3C) or the NRG1 Type III antibody that was raised against a region within the C-terminal domain of NRG1 Type III (Figure 3D) did not decrease the detection of these NRG1 isoforms, further supporting the specificity for the custom antibody against an N-terminal fragment of NRG1 Type III. Immunoblotting for β-actin indicated that similar amounts of protein were present in each sample. Unfortunately, we were unable to detect the presence of the 165 kDa unprocessed NRG1 with any antibodies used in the current work.

**Figure 4:**
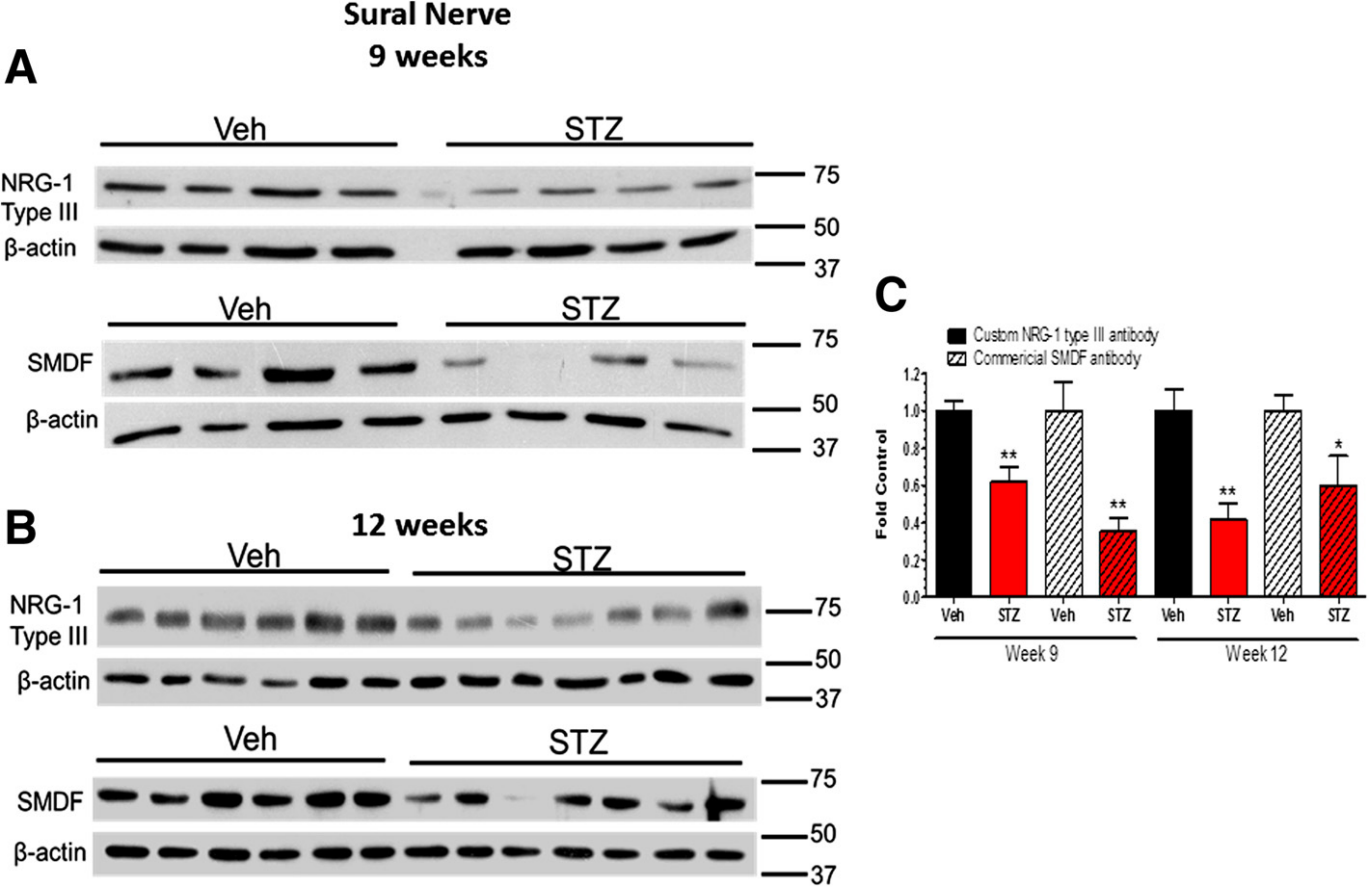
**NRG1 Type III was decreased in sural nerve after 9 and 12 weeks of diabetes.** Mice were treated with vehicle (Veh) or STZ and sural nerves were isolated at 9 **(A)** or 12 **(B)** weeks after the induction of diabetes (n = 6–7 per group). Protein lysates were prepared and NRG1 Type III level was determined by immunoblot using the two antibodies. **(C)**: Bands were quantified, NRG1 Type III levels were normalized to β-actin and expressed as a fold of the levels in control sural nerves (*, p < 0.05 versus Veh; **, p < 0.01 versus Veh).

**Figure 5:**
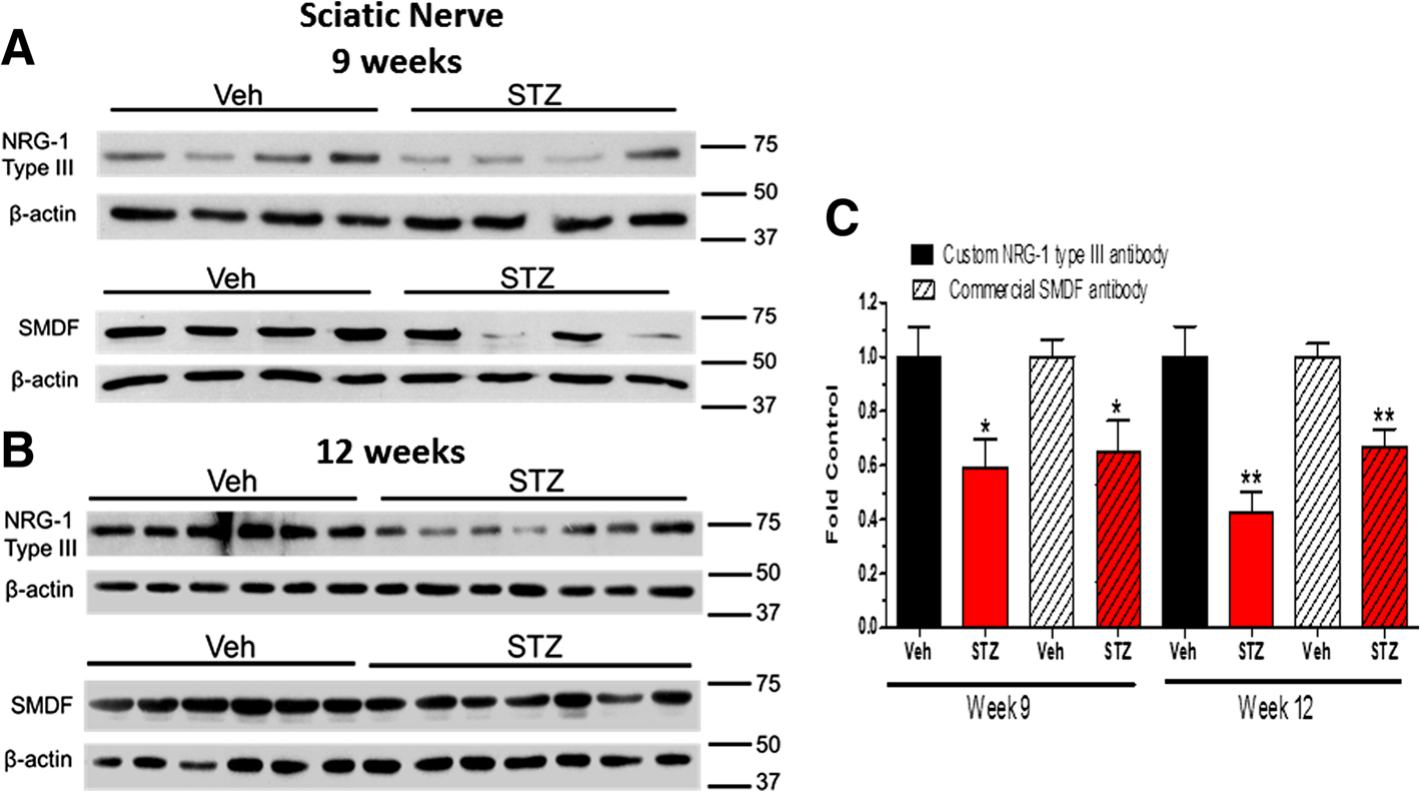
**NRG1 Type III was decreased in sciatic nerve after 9 and 12 weeks of diabetes.** Mice were treated with vehicle (Veh) or STZ and sciatic nerves were isolated at 9 **(A)** or 12 **(B)** weeks after the induction of diabetes (n = 6–7 per group). Protein lysates were prepared and NRG1 Type III level was determined by immunoblot using the two antibodies. **(C)**: Bands were quantified, NRG1 Type III levels were normalized to β-actin and expressed as a fold of the levels in control sciatic nerves (*, p < 0.05 versus Veh; **, p < 0.01 versus Veh).

**Figure 6:**
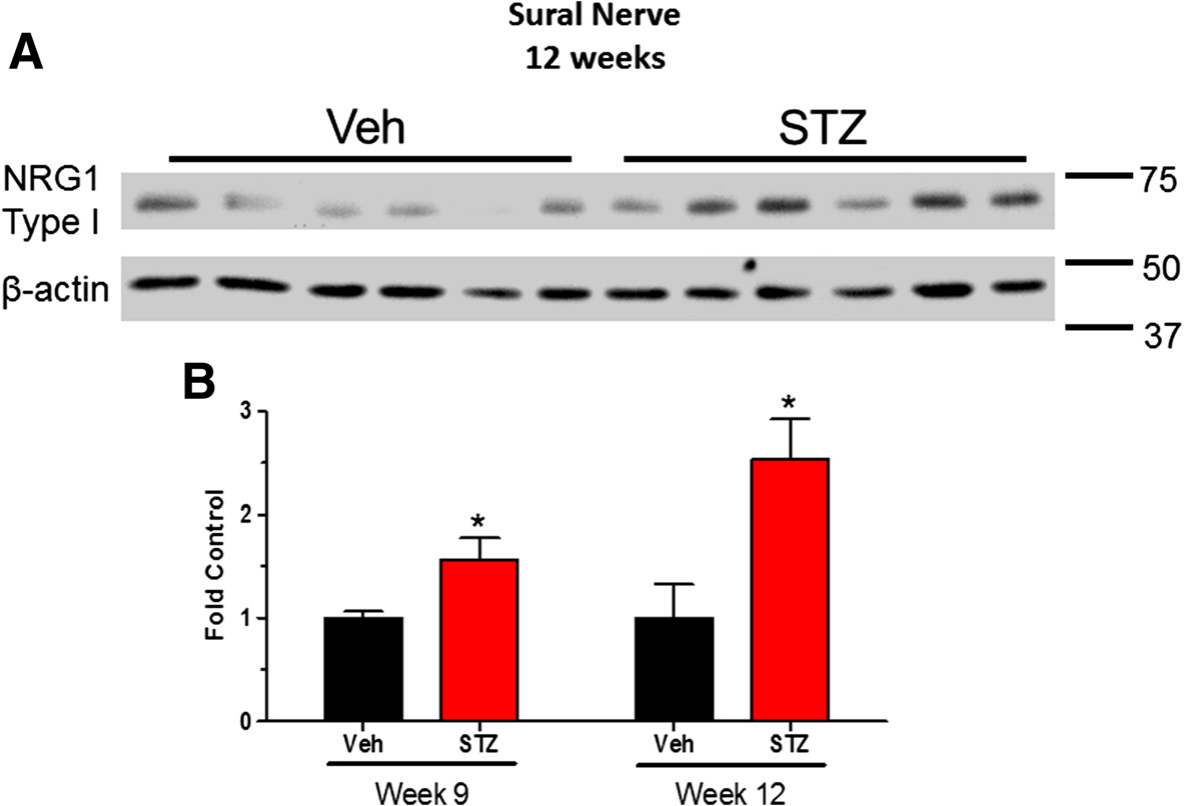
**NRG1 Type I was increased in sural nerves after 9 and 12 weeks of diabetes. (A)**: Sural nerves were isolated from 9 and 12-week control and diabetic mice (n = 6–7 per group). Protein lysates were prepared and NRG1 Type I levels were determined by immunoblot using the Abcam NRG1 Type I antibody. **(B)**: Bands were quantified, NRG1 Type I levels were normalized to β-actin and expressed as a fold of the levels in control sural nerves (*, p < 0.05 versus Veh).

After 9 weeks (Figure 4A) and 12 weeks (Figure 4B) of diabetes, immunoblot analysis with both antibodies showed a significant decrease in the 75 kDa and 65 kDa NRG1 Type III bands in sural nerve (Figure 3D). Similarly, the expression of NRG1 Type III was also decreased in sciatic nerve after 9 and 12 weeks of diabetes (Figure 5A-C). Given the role of axonal NRG1 Type III in promoting SC differentiation, these data suggest that diabetes may compromise myelination by decreasing NRG1 Type III levels. However, a decrease in NRG1 Type III would not be anticipated to stimulate Erb B2 activity in peripheral nerve. Since NRG1 Type I can stimulate SC dedifferentiation [17], we also investigated whether altered expression of this isoform is associated with DPN. After 9 weeks of diabetes, the level of NRG1 Type I was modestly increased in sural nerve but this response was more robust after 12 weeks (Figure 6A and B). However, the levels of NRG1 Type I in diabetic sciatic nerve remained unchanged at both time points (data not shown). These data suggest that diabetes can differentially alter the expression of NRG1 isoforms, especially in the largely sensory sural nerve.

### Diabetes decreases erbin expression and increases p42/p44 MAPK activity

**Figure 7:**
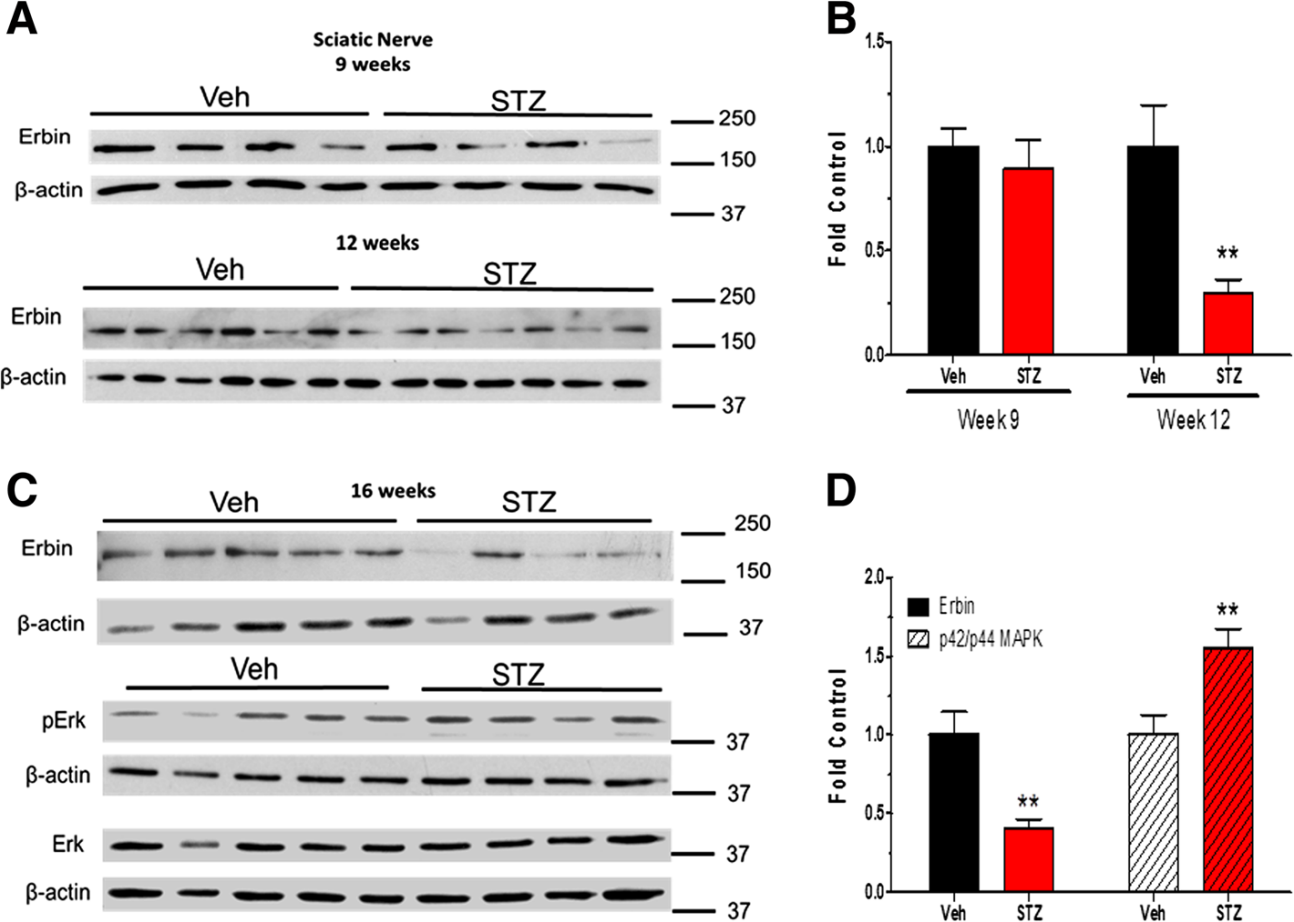
**Erbin was decreased and p42/p44 MAPK activity enhanced in diabetic sciatic nerve.** Sciatic nerves were isolated from 9, 12 **(A)** and 16-week **(C)** control and diabetic mice (n = 8–9 per group). Protein lysates were prepared and Erbin levels and p42/p44 MAPK (pErk) levels were determined by immunoblot. Quantification demonstrated a significant decrease of Erbin levels **(B)** and an increase in p42/p44 MAPK activation at 16 weeks **(D)**. (*, p < 0.05 versus Veh; **, p < 0.01 versus Veh).

**Figure 8:**
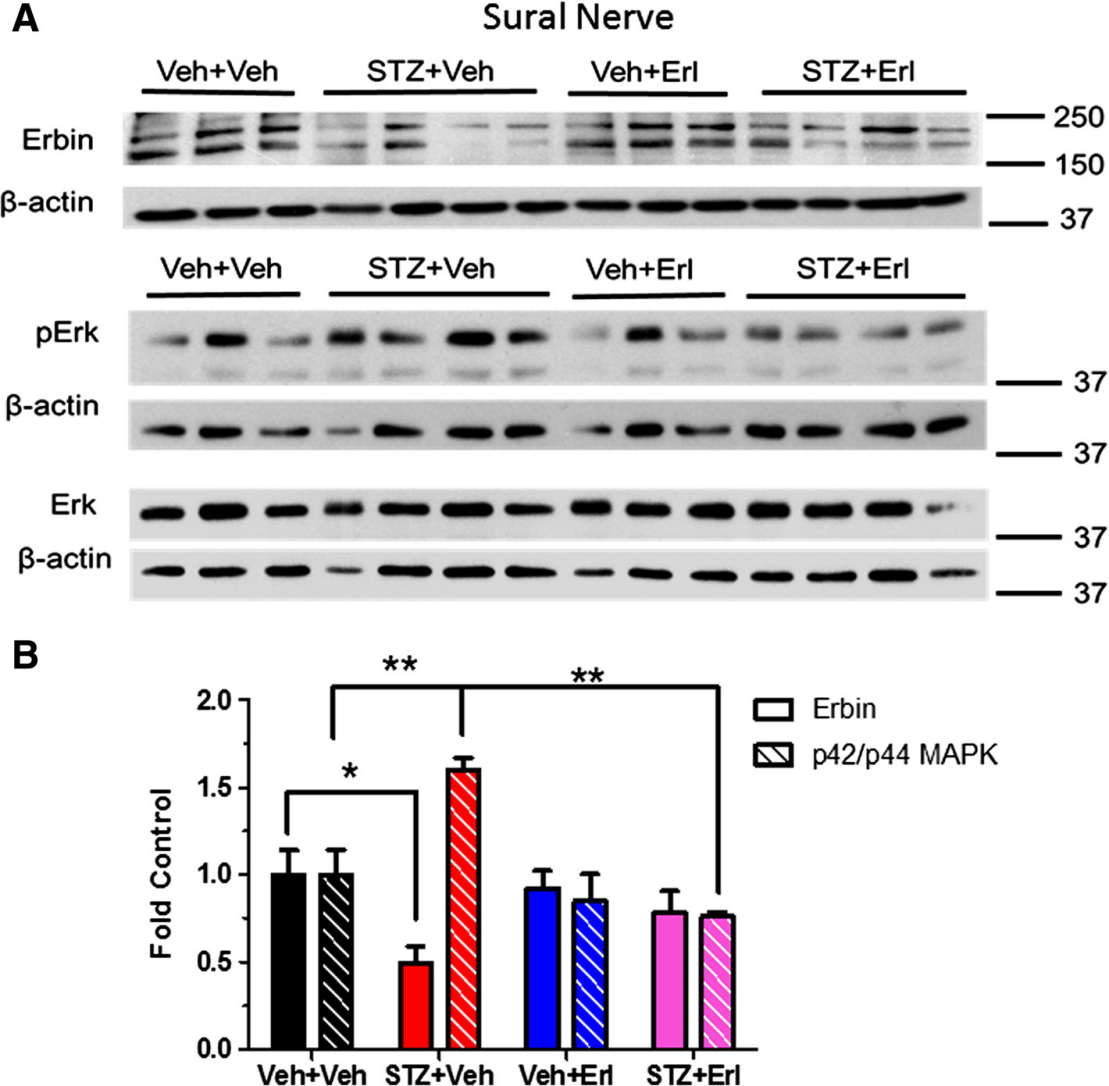
**Inhibition of Erb B2 with erlotinib suppressed diabetes-induced p42/p44 MAPK pathway activation in sural nerves. (A)**: Sural nerves were isolated from vehicle or erlotinib-treated control and diabetic mice (n = 3–4 per group) at week 21. Protein lysates were prepared and erbin levels and p42/p44 MAPK (pErk) levels were determined by immunoblot. **(B)**: Quantification demonstrated a significant decrease of erbin and an increase in p42/p44 MAPK activation in vehicle-treated diabetic mice. Erlotinib treatment suppressed p42/p44 MAPK activation in erlotinib-treated diabetic mice (*, p < 0.05; **, p < 0.01).

Erbin is an Erb B2 interacting protein that can function as a negative regulator of receptor signaling, in part by inhibiting p42/p44 MAPK activity [28]. Thus, a decrease in erbin expression may also contribute to potential dysregulation of Erb B2 signaling in diabetic nerve. Diabetic sciatic nerve showed a decreased expression of erbin at 12 and 16 weeks (Figure 7A-D). However, enhanced activity of p42/p44 MAPK pathway was only detected in 16-week diabetic sciatic nerves (Figure 7D). Though it was difficult to detect erbin in sural nerve, its level also decreased after 21 weeks of diabetes and this correlated with an increase in phosphorylation of p42/p44 MAPK. Treatment with erlotinib treatment significantly decreased the extent of MAPK phosphorylation (Figure 8A and B).

## Discussion

Our prior work was the first to suggest that altered signaling via Erb B2 can contribute to the onset of DPN [21] but it was unclear if changes in Erb B2 signaling may be associated with an altered expression of NRG1. The current study extends this work and is the first to demonstrate that diabetes can alter the expression of NRG1 isoforms in peripheral nerves. While we observed a reduction in NRG1 Type III levels in both diabetic sciatic and sural nerves, the level of NRG1 Type I increased in sural nerve after 9–12 weeks of diabetes. Since inhibition of Erb B2 with erlotinib partially reversed the sensory deficits associated with prolonged diabetes, the enhanced expression of NRG1 Type I in diabetic sural nerves might be sufficient to elevate Erb B2 receptor activation and contribute to the progression of DPN. Moreover, the decreased NRG1 Type III and increased NRG1 Type I expression observed in diabetic sural nerve (but not in diabetic sciatic nerves) implies that distal and more thinly myelinated fibers may be more sensitive to early disruption of NRG1 signaling. This would be consistent with recent results showing a greater severity of oxidative stress in distal sural nerve compared to sciatic nerve [29].

NRG1 Type III is a membrane-anchored precursor protein which needs to be proteolytically processed to become an active signaling molecule. The β-secretase BACE1 (β-site amyloid precursor protein cleaving enzyme 1) and the α-secretase TACE (tumor necrosis factor-α-converting enzyme) are two enzymes that process NRG1. NRG1 Type III processed by BACE1 produces a membrane-anchored, N-terminal fragment that promotes myelination [30], whereas TACE cleaves NRG1 Type III in the EGF-like domain and inactivates it, resulting in hypomyelination [31]. BACE cleavage also produces the C-terminal fragment which can be degraded by γ-secretase. Though the C-terminal fragment is not necessary for myelination [32], it may be translocated to the nucleus of neurons and can repress apoptosis and promote survival [33]. Diabetes-induced changes in the expression of NRG1 Type III produced by BACE cleavage were verified using two antibodies which targeted either the CRD located within the ~75 kDa N-terminal fragment or an epitope within the C-terminal fragment. Interestingly, we did not consistently observe the presence of unprocessed NRG1 Type III in nerves from either control or diabetic mice. This suggests that the decrease in the expression of the N- and C-terminal fragments of NRG1 Type III were not due to a decrease in proteolytic processing. Thus, changes in the rate of transcription and/or translation may contribute to the decrease in NRG1 Type III. However, we also observed an increase in NRG1 Type I in diabetic sural nerve. Since both isoforms are the product of alternative splicing of a single transcript, it is possible that diabetes-induced changes in mRNA processing may alter the isoform expression pattern. In this regard, diabetes has been shown to alter expression of transcriptional variants of the *Slo* gene that may contribute to erectile dysfunction [34]. However, diabetes may have tissue specific effects on the expression of NRG1 isoforms. For example, the levels of NRG1 Type I were decreased in diabetic rats with cardiomyopathy [35] and impaired signaling through the NRG1/Erb B cassette may contribute to the pathogenesis of diabetic cardiomyopathy, increasing susceptibility to heart failure [36].

The degeneration of sensory neurons in DPN is clearly associated with an alteration in neurotrophic support and disrupted NRG-1/Erb B2 signaling, presumably in SCs, may be interconnected with altered neurotrophism. BDNF is released from SCs, is decreased in diabetic rats [37] and treatment with BDNF prevented nerve conduction slowing and damage to large motor fibers [38]. A clear relationship exists between BDNF and NRG1 signaling since BDNF can also stimulate the secretion of soluble forms of NRG1 [39] whereas transgenic inhibition of endogenous Erb B2 via expression of a dominant-negative Erb B4 in non-myelinating SCs was sufficient to decrease the expression of BDNF [14]. Though it remains unclear whether changes in BDNF levels may have contributed to the altered expression of NRG1 isoforms observed in diabetic nerve in the current study, elucidating the effect of diabetes on the activity of neurotrophins and neuregulins in dedifferentiating and regenerating SCs may provide fundamental insight into the potential for pharmacologically regulating Erb B2 signaling at specific disease stages to improve nerve function. Lastly, recent data also suggests that axonal expression of NRG1 Type III can negatively regulate the expression of SC-derived NRG1 Type I [40]. Although these results were obtained in the context of a decrease in NRG1 Type III due to axonal loss following nerve crush, axonal loss is not a hallmark of the rather early stage of DPN modeled in our study. Thus, a diabetes-induced alteration in the expression of NRG1 Type III without frank axonal loss may be sufficient to promote the expression of NRG1 Type I. However, additional work is required to determine if the negative regulation of NRG1 Type I expression by axonal NRG1 Type III may be recapitulated following a peripheral nerve injury that is solely metabolic.

Erbin functions as an adapter protein that binds to Erb B2 and it plays an important role in myelination since erbin knockout mice have a decrease in Erb B2 levels and NRG1-induced myelination [20,41]. Erbin also serves as a negative regulator of p42/p44 MAPK signaling by disrupting the interaction between Ras and Raf [28,42]. Consistent with this relationship, diabetes decreased erbin levels in sciatic and sural nerve and this correlated with an increase in the activity of p42/p44 MAPK. Although erlotinib treatment inhibited the activation of p42/p44 MAPK in sural nerve, without increasing erbin expression, it is not possible to directly link this change in MAPK activity to the improved sensory endpoints following erlotinib treatment since the drug would be expected to blunt all signaling through Erb B2. Though activation of the MAPK pathway has been associated with demyelination [18,43], myelin loss is not a hallmark of DPN in rodent models. Therefore, the activation of this pathway is either of insufficient magnitude and duration to promote demyelination in rodent nerve or contributes to other aspects of DPN. However, the contribution of p42/p44 MAPK activity to DPN in both rodent and human DPN is unclear and these enzymes showed variable activation in sural nerve biopsies obtained from diabetic patients undergoing amputations [44].

## Conclusions

In summary, our data support that diabetes may alter Erb B2 signaling in peripheral nerve by altering the balance in NRG1 isoform expression and decreasing the expression of erbin, an adapter protein that can function as a negative regulator of p42/p44 MAPK signaling via Erb B2. Though a limitation of our study is that we can not ascertain if changes in NRG1 isoforms are necessary or sufficient to contribute to DPN, altered signaling through the NRG1-Erb B2 ligand/receptor pair may contribute to dysfunction of both myelinated and unmyelinated fibers since diabetic mice treated with erlotinib exhibited an improvement in both mechanical and thermal sensitivity. Given the complex role of neuregulins in controlling both myelination and demyelination, these data suggest that an altered neuregulinism may contribute to myelin pathologies that develop in human DPN.

## Methods

### Reagents and antibodies

Streptozotocin (STZ) was obtained from Sigma-Aldrich (St. Louis, MO). *N*-(3-ethynylphenyl)-6,7-bis(2-methoxyethoxy)-4-quinazolinamine (erlotinib) was provided by OSI Pharmaceuticals (Melville, NY). NRG1 Type III polyclonal antibody was produced in rabbits by Chi Scientific (Maynard, MA) using the CIAGLKWVFVDKIFEYDSPTHL peptide of NRG1 Type III as the immunizing antigen [12]. CIAGLKWVFVDKIFEYDSPTHL peptide was synthesized by Selleck Chemicals (Houston, TX). A commercial NRG1 Type III antibody (SMDF C-16) and horseradish peroxidase–conjugated secondary antibodies were obtained from Santa Cruz Biotechnology (Santa Cruz, CA). NRG1 Type I (ab27303) and erbin (ab55930) antibodies were purchased from Abcam (Cambridge, MA). Other antibodies and their sources were: β-actin antibody (#691002, MP Biomedicals, Solon, OH); phospho-MAPK 1/2 (#9101) and total MAPK 1/2 (#9102) antibodies (Cell Signaling, Boston, MA).

### Induction of diabetes and drug treatment

Swiss Webster mice were obtained from Harlan Laboratories (Indianapolis, IN) and eight-week old mice were rendered diabetic with freshly prepared STZ given by intraperitoneal injection at 100 mg/kg for two consecutive days. One week after the last injection, mice were fasted for 6 hrs and blood was obtained from the tail vein. Mice with a fasting blood glucose ≥ 290 mg/dl (16 mmol/l) were deemed diabetic (One-Touch Ultra glucometer). All animals were maintained on a 12-h light/dark cycle with ad libitum access to water and Purina diet 5001 rodent chow. To inhibit Erb B2, erlotinib was dissolved in 0.1 mM Captisol and given at 25 mg/kg via intraperitoneal injection either once or twice per week. Final levels of fasting blood glucose and glycated hemoglobin (HbA1c, A1C Now^+^) were determined immediately before euthanizing the animals. All animal procedures were performed in accordance with protocols approved by the Institutional Animal Care and Use Committee and in compliance with standards and regulations for care and use of laboratory rodents set by the National Institutes of Health.

### Measure of sensory hypoalgesia and nerve conduction velocities (NCV)

Mechanical and thermal sensitivity were measured as previously described using a Dynamic Plantar Aesthesiometer (Stoelting, Wood Dale, IL) and a Hargreaves Analgesiometer, respectively [26]. Withdrawal force (grams) and paw withdrawal latency (seconds) are the average of three to four trials taken from alternating feet with 5-min periods between testing. Motor and sensory nerve conduction velocities were assessed in deeply anesthetized mice as previously detailed [21]. Whole body (rectal probe) and near nerve temperatures (subcutaneous probe) of anesthetized mice were maintained at 36–37°C using a heating pad and heat lamp.

### Immunoblot analysis

Peripheral nerves were homogenized in 0.2-ml lysis buffer (50 mmol/l Tris–HCl, pH 7.5, 1 mmol/l EDTA, 1% Nonidet P-40, 0.5% deoxycholate, 1 mmol/l Na_3_VO_4_, 150 mmol/l NaCl, 0.5 mmol/l sodium molybdate, 40 mmol/l NaF, 10 mmol/l β-glycerophosphate, and 1X Complete Protease inhibitors) (Roche Diagnostics) with the aid of a Polytron fitted with a micro tissue tearor. Cell debris was sedimented at 10,000 × *g* for 5 min at 4°C, and the protein concentration of the supernatant was determined. Proteins were separated by SDS-PAGE and transferred onto nitrocellulose for immunoblot analyses. Immunoblots were quantified by densitometry with the aid of ImageJ software and the level of the proteins of interest was normalized to β-actin unless otherwise stated. Changes in protein expression are expressed as a percent of the control values.

### Peptide competition assay

Sciatic nerves from untreated adult mice were homogenized in lysis buffer, proteins were separated by SDS-PAGE and transferred onto nitrocellulose for immunoblot analyses. The various NRG1 antibodies were preabsorbed with buffer or the immunizing peptide at a 1:50, 1:100 or 1:200 mass ratio by incubation for 1 hr at 25°C prior to their use for detecting NRG1 isoforms in the nerve lysate by immunoblot analysis.

### Immunohistochemistry analysis

The integument of the plantar surface of both hind paws was dissected and placed in Zamboni’s fixative overnight. Tissues were then rinsed in PBS at 4°C, cryoprotected in 30% sucrose overnight, embedded in OCT, frozen on dry ice, and stored at -80°C. Frozen tissues were sectioned at 30 μm, placed on Fisherbrand Superfrost Plus microscope slides and stored at -80°C. Immunohistochemistry was performed using the Vectastain Elite ABC-Peroxidase kit for rabbit IgG (Vector Laboratories, Burlingame, CA) and an anti PGP 9.5 antibody (AbD Serotec, Oxford, UK). Sections were then counterstained by NovaRED peroxidase substrate solution (Vector Laboratories, Burlingame, CA) and with hematoxylin. A total of twelve images per animal were captured on a Zeiss LSM 510 Meta confocal microscope for quantification. Single nerve fibers crossing the dermal/epidermal junction were counted from each image. The IENF density was calculated by the number of fibers counted divided by the length of the dermal/epidermal junction (fibers/mm).

### Statistical analysis

Data are presented as means ± SEM. After verifying equality of variance, differences between treatments were determined using a one-way or two-way ANOVA. Differences between group means were ascertained using Tukey’s test.

## Abbreviations

BACE1: β-site amyloid precursor protein cleaving enzyme 1; BDNF: Brain derived-nerurotrophic factor; CRD: Cysteine-rich domain; DPN: Diabetic peripheral neuropathy; EGF: Epidermal growth factor; EGFR: Epidermal growth factor receptor; Erbin: Erb B2-interacting protein; IENFD: Intraepidermal nerve fiber density; MAPK: Mitogen-activated protein kinase; MNCV: Motor nerve conduction velocity; NGF: Nerve growth factor; NRG1: Neuregulin-1; PGP 9.5: Protein gene product 9.5; SC: Schwann cell; SMDF: Sensory and motor neuron derived factor; SNCV: Sensory nerve conduction velocity; STZ: Streptozotocin; TACE: Tumor necrosis factor-α-converting enzyme.

## Competing interests

The authors declare that they have no competing interests.

## Authors’ contributions

PP performed the study and wrote the manuscript; RTD designed the research, wrote and edited the manuscript. Both authors read and approved the final manuscript.
